# An audit into the management of COVID-19 in a high security psychiatric hospital

**DOI:** 10.1192/bjo.2021.224

**Published:** 2021-06-18

**Authors:** Elliott Carthy, Samrat Sengupta

**Affiliations:** 1 Warneford Hospital; 2 Broadmoor Hospital, Crowthorne Berks

## Abstract

**Aims:**

Comprehensive and timely data collection during a pandemic is crucial in developing guidelines and policy as well as evaluating their effectiveness. In turn, this will improve planning for future incidents. While this is being undertaken at a national level by Public Health England, more specific information as it relates to psychiatric care is important in understanding the neuropsychiatric, psychological and social effects of the pandemic. The management of patients with COVID-19 presents a unique challenge in inpatient psychiatry settings both in terms of diagnosis and treatment. This is perhaps greater still in forensic settings due to the increased risk of violence and aggression. This audit aimed to firstly assess the consistency of local practice to national guidance from Public Health England. Secondly, it aimed to describe the clinical management of suspected and confirmed cases of COVID-19 in this high security forensic hospital and how readily broad, national guidance can be implemented in this unique setting. We present an audit with three cycles, one from each wave of COVID-19 in England during 2020.

**Method:**

This was a retrospective audit in a high secure forensic psychiatry hospital in the United Kingdom, into the investigation and management of suspected and confirmed cases of COVID-19 compared to national guidelines from Public Health England. It includes three cycles, one undertaken in each national wave of COVID-19 in England in 2020.

**Result:**

Ten patients have been included in cycle 1, 12 in cycle 2 and 21 in cycle 3 as those where COVID-19 was a considered diagnosis. SARS-CoV-2 was detected in one patient in cycle 1 and 12 patients in cycle 3. All patients recovered, most of whom remained on-site with supportive care in self-isolation on a dedicated ward for positive cases. Three patients required additional treated with oral antibiotics and dexamethasone, one of whom required admission to the local general medical hospital for continuous supplemental oxygen.

**Conclusion:**

This is the first study to describe the management of the novel COVID-19 pandemic in a high security forensic psychiatry hospital and how readily national guidance can be implemented in this unique setting. Hospital practice at identifying suspected cases and the management of confirmed cases of COVID-19 was shown to be consistent with national guidance. It also allowed for clinicians to exercise their judgement about testing for atypical cases and for repeat testing where appropriate.

